# The association between nutritional risk and contrast-induced acute kidney injury in patients undergoing coronary angiography: a cross-sectional study

**DOI:** 10.1186/s12937-022-00810-z

**Published:** 2022-09-16

**Authors:** Duanbin Li, Zhezhe Chen, Wujian He, Li Lin, Tian Xu, Hangpan Jiang, Lu Liu, Guosheng Fu, Zhaoyang Chen, Wenbin Zhang

**Affiliations:** 1 Department of Cardiology, Sir Run Run Shaw Hospital, College of Medicine, Zhejiang University, No 3 East of Qingchun Road, Hangzhou, 310000 Zhejiang Province China; 2 Key Laboratory of Cardiovascular Intervention and Regenerative Medicine of Zhejiang Province, Hangzhou, 310000 Zhejiang Province China; 3 Department of Cardiology, Hangzhou Red Cross Hospital, No 208 Huancheng East Road, Hangzhou, 310000 Zhejiang Province China; 4 Department of Cardiology, Union Hospital, Fujian Medical University, No 29 Xin-Quan Road, Fuzhou, 350001 Fujian Province China; 5 Department of Cardiology, The Fourth Affiliated Hospital, College of Medicine, Zhejiang University, No 1 Shangcheng Avenue, Yiwu, 322000 Zhejiang Province China

**Keywords:** Nutritional risk screening 2002, Controlling nutritional status, Prognostic nutritional index, Geriatric nutritional risk index, Contrast-induced acute kidney injury

## Abstract

**Background:**

Nutritional risk is prevalent in various diseases, but its association with contrast-induced acute kidney injury (CI-AKI) remains unclear. This study aimed to explore this association in patients undergoing coronary angiography (CAG).

**Methods:**

In this retrospective cross-sectional study, 4386 patients undergoing CAG were enrolled. Nutritional risks were estimated by nutritional risk screening 2002 (NRS-2002), controlling nutritional status (CONUT), prognostic nutritional index (PNI), and geriatric nutritional risk index (GNRI), respectively. CI-AKI was determined by the elevation of serum creatinine (Scr). Multivariable logistic regression analyses and receiver operator characteristic (ROC) analyses were conducted. Subgroup analyses were performed according to age (< 70/≥70 years), gender (male/female), percutaneous coronary intervention (with/without), and estimated glomerular filtration rate (< 60/≥60 ml/min/1.73m^2^).

**Results:**

Overall, 787 (17.9%) patients were diagnosed with CI-AKI. The median score of NRS-2002, CONUT, PNI, and GNRI was 1.0, 3.0, 45.8, and 98.6, respectively. Nutritional risk was proven to be associated with CI-AKI when four different nutritional tools were employed, including NRS-2002 ([3–7 vs. 0]: odds ratio [95% confidence interval], OR [95%CI] = 4.026 [2.732 to 5.932], *P* < 0.001), CONUT ([6–12 vs. 0–1]: OR [95%CI] = 2.230 [1.586 to 3.136], *P* < 0.001), PNI ([< 38 vs. ≥52]: OR [95%CI] = 2.349 [1.529 to 3.610], *P* < 0.001), and GNRI ([< 90 vs. ≥104]: OR [95%CI] = 1.822 [1.229 to 2.702], *P* = 0.003). This is consistent when subgroup analyses were performed. Furthermore, nutritional scores were proved to be accurate in predicting CI-AKI (area under ROC curve: NRS-2002, 0.625; CONUT, 0.609; PNI, 0.629; and GNRI, 0.603).

**Conclusions:**

Nutritional risks (high scores of NRS-2002 and CONUT; low scores of PNI and GNRI) were associated with CI-AKI in patients undergoing CAG.

**Supplementary Information:**

The online version contains supplementary material available at 10.1186/s12937-022-00810-z.

## Background

Coronary artery disease (CAD) has been the primary cause of mortality around the world [1, 2]. For decades, the development of new technologies has dramatically changed this situation, especially the widespread use of coronary angiography (CAG) and percutaneous coronary intervention (PCI) [3]. However, with the increasing use of CAG/PCI, contrast-induced acute kidney injury (CI-AKI) has emerged as a new challenge [4].

CI-AKI is one of the most common complications after CAG/PCI and the third leading cause of iatrogenic renal dysfunction [5, 6]. The European Society of Urogenital Radiology (ESUR) defines CI-AKI as a dramatic increase of serum creatinine (Scr) ≥ 44 μmol/L (0.5 mg/dL) or ≥ 25% within 72 h following the exposure to contrast [7, 8]. Previous studies reported a CI-AKI incidence of approximately 15% in patients undergoing CAG/PCI, which can further increase to 50% in high-risk individuals [9, 10]. Patients diagnosed with CI-AKI had higher risks of subsequent mortality, prolonged hospitalization stays, and increased hospitalization costs [11]. Moreover, many risk factors for CI-AKI have been identified including older age, renal dysfunction, cardiac dysfunction, diabetes, anemia, hemodynamic instability, serum klotho protein, the type of contrast agent, and absence of statins use [12–16].

Nutritional risk is prevalent in a variety of diseases, especially in age-related degenerative diseases [17]. Increased nutritional risks are related to poorer clinical prognoses in cardiovascular disease [18]. Serum albumin, body mass index, and blood lipids were traditionally used to assess nutritional status, while the stability and comprehensiveness of these indicators are inadequate [19]. Nutritional screening tools can be easily applied to provide a more comprehensive and objective assessment of nutrition risks. Nutritional risk screening 2002 (NRS-2002), controlling nutritional status (CONUT), prognostic nutritional index (PNI), and geriatric nutritional risk index (GNRI) are four different well-established nutritional screening tools in clinical practice [20–22]. Higher scores of NRS-2002 and CONUT, and lower scores of PNI and GNRI all indicate an underlying nutritional risk.

Despite the prevalence of nutritional risk, its relationship with CI-AKI remains unclear. Therefore, we conducted the current study to explore the relationship between nutritional risks and CI-AKI by using four different nutritional screening tools in patients undergoing CAG or PCI.

## Methods

### Study design

This is a retrospective cross-sectional study. Patients undergoing CAG or PCI were eligible for screening from January 2009 to December 2019 at Sir Run Run Shaw Hospital and its medical consortium hospitals. Fig. S1 shows the flow chart of the patient selection. The following subjects were included: a. patients undergoing CAG/PCI; b. the data of NRS-2002, CONUT, PNI, and GNRI scores can be retrospectively calculated or obtained; c. Scr levels were assessed on admission and within 72 hours after CAG/PCI; d. data of demographic, laboratory testing, CAG/PCI, and medication was available for analysis. The following subjects were excluded: a. repeated exposure to contrast agent during hospitalization; b. subjects with end-stage renal diseases requiring hemodialysis; c. pre-procedure estimated glomerular filtration rate (eGFR) under 15 ml/min/1.73m^2^; d. active malignant tumor on admission; e. patients in shock, pregnancy, or lactation. Eventually, a total of 4386 patients were enrolled.

The Strengthening the Reporting of Observational Studies in Epidemiology (STROBE) guideline was followed to report this study [23]. The ethical approval was obtained from the Medical Ethical Review Committee of Sir Run Run Shaw Hospital (20201217–36).

### The assessment of nutritional risks

The nutritional risk was estimated by four different nutritional risk screening tools, including NRS-2002, CONUT, PNI, and GNRI. Higher scores of NRS-2002 and CONUT, lower scores of PNI and GNRI indicate an underlying nutritional risk. All patients were routinely screened for NRS-2002 by a trained physician on admission. CONUT, PNI, and GNRI were retrospectively calculated based on pre-procedure laboratory testing data. Four nutritional screening tools are depicted as follows.

NRS-2002 contains three components: disease severity, impaired nutritional status, and age, giving a total score of 0–7. The impaired nutritional status is determined by three variables: reduced food intake, unintentional weight loss, and body mass index. The patient with an NRS-2002 score ≥ 3 was considered malnourished [24].

CONUT is a nutritional screening tool with a total score of 0–12, which includes three laboratory indicators: albumin level, lymphocyte count, and total cholesterol [25]. The scoring system for CONUT was represented in Table S1.

PNI uses albumin and lymphocyte count to assess nutrition risks, which is estimated by the formula: PNI = 10 × serum albumin (g/dl) + 0.005 × total lymphocytes (/μl) [18].

GNRI is a tool for hospitalized elderly patients and is calculated by the formula: GNRI = 1.489 × serum albumin (g/L) + 41.7 × (weight/ideal weight) [26]. Ideal weight was estimated according to body height (H, cm) from the Lorentz equations as follows: ideal weight for men = H-100-[(H-150)/4]; ideal weight for women = H-100-[(H-150)/2.5] [26]. The weight/ideal weight ratio is set to one when its actual ratio is greater than one.

### The definition of CI-AKI

Scr levels were routinely assessed on admission and within 72 hours after CAG/PCI. The proportion of Scr elevation was calculated by the formula: (post-procedure maximum Scr - pre-procedure Scr) / pre-procedure Scr × 100%, and post-procedure Scr was estimated within 72 hours after CAG/PCI. CI-AKI was determined by the elevation of Scr levels according to the diagnostic criteria of ESUR, including a. an increase in Scr by more than 44 μmol/L (0.5 mg/dl) or 25%; b. within 72 h of intravascular contrast injection; c. no alternative etiology [7, 8].

### Statistical analysis

Continuous variable with normal distribution was displayed by the mean ± standard deviation (SD) and compared by independent sample Student’s t-test. Continuous variable with non-normal distribution was displayed by median (interquartile range) and compared by Kruskal-Wallis test. The categorical variable was displayed by count (proportion) and compared using the Chi-square test.

The correlation analysis was performed by using Spearman rank-order correlation and visualized by a correlation matrix using the R package ‘corrplot’. The association between nutritional scores and the proportion of Scr elevation was visualized by the scatter plots with linear fits using the R package ‘ggplot2’ and verified by multivariable linear regression analyses. The association between nutritional scores and CI-AKI was estimated by multivariable logistic regression analyses and visualized by restricted cubic spline models. *P* value for linear trend (*P* for trend) was calculated by treating categorical variables as ordinal in logistic regression models. Multivariable regression analyses adjusted the underlying confounding factors for CI-AKI, which was identified by previous studies [27, 28]. The sample size was evaluated using a common rule of thumb. More than 20 events per variable (total events = 787) have been achieved in regression analyses, which indicates the reliability of the results. Receiver operating characteristic (ROC) analyses were employed to assess predictive values of nutritional scores for CI-AKI using the R package ‘ROCit’. Finally, subgroup analyses were conducted according to age (< 70 or ≥ 70 years), gender (male or female), PCI (with or without), and eGFR (< 60 or ≥ 60 ml/min/1.73m^2^). Tests for interaction (nutritional categories × subgroup stratification) were performed by the likelihood ratio test. Significance was determined by a two-tailed *P* value < 0.05. Statistical analyses were conducted by using SPSS software version 22.0 (SPSS Inc., Chicago, IL, USA) and R version 3.5.1 (The R Foundation for Statistical Computing, Vienna, Austria).

## Results

### Study participants

**Fig. 1:**
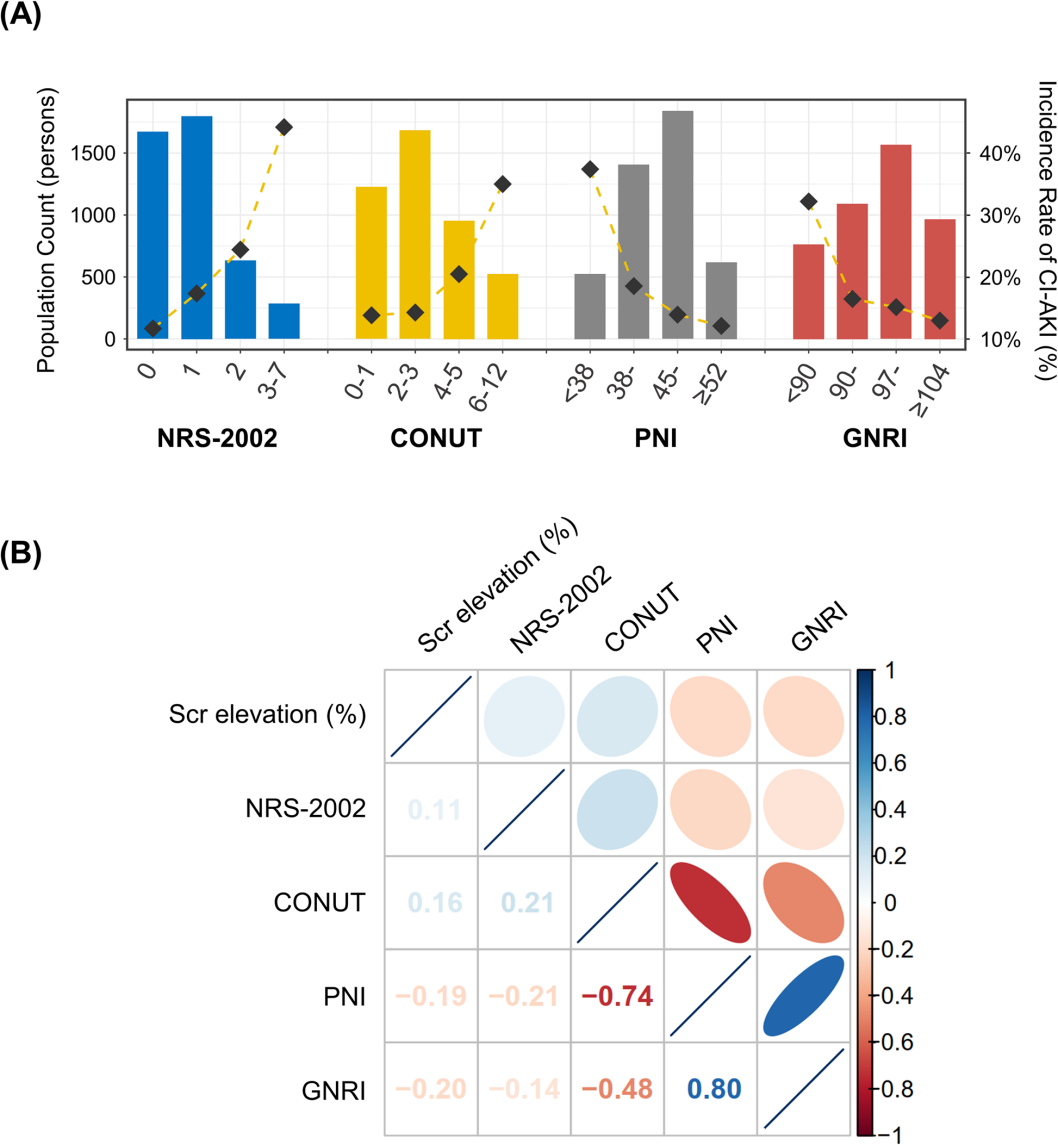
The population distribution and correlation matrix. **A** The distribution of nutritional scores and the CI-AKI incidence. Bar plots depict the population distribution according to categories of nutritional scores. The dashed line chart depicts the change of the incidence of CI-AKI. Left axis, population count (persons); right axis, the incidence rate of CI-AKI (%); (**B**) Correlation matrix of the proportion of Scr elevation and nutritional scores. Coefficients of spearman rank-order correlations are displayed (all *P* values < 0.001). A higher correlation is represented by lower transparency and narrower ellipses. Blue indicates positive correlation and red indicates negative correlation. NRS-2002 indicates nutritional risk screening 2002; CONUT, controlling nutritional status; PNI, prognostic nutritional index; GNRI, geriatric nutritional risk index; Scr, serum creatinine; CI-AKI, contrast-induced acute kidney injury

Overall, 4386 subjects undergoing CAG/PCI were included. The age was 67.1 ± 10.8 years old, 2895 (66.0%) patients were male, and 1993 (45.4%) patients underwent PCI (Table 1). Among these, 787 (17.9%) subjects were diagnosed with CI-AKI after intravascular contrast injection. Patients with CI-AKI have a worse nutritional status, including higher scores of NRS-2002 (1.0 [1.0, 2.0] vs. 1.0 [0.0, 1.0], *P* < 0.001), higher scores of CONUT (3.0 [2.0, 5.0] vs. 3.0 [1.0, 4.0], *P* < 0.001), lower scores of PNI (43.5 [38.0, 48.2] vs. 46.32 [42.5, 50.1], *P* < 0.001), and lower scores of GNRI (95.9 [88.4, 101.7] vs. 99.0 [93.5, 103.6], *P* < 0.001). The incidence of CI-AKI increases gradually with poor nutritional status (Fig. 1A).

**Table 1:** Baseline characteristics

	Overall	CI-AKI		*P* value
Characteristics	(*n* = 4386)	No (*n* = 3599)	Yes (*n* = 787)	
**Demographic features**				
Age, years old	67.1 ± 10.8	66.6 ± 10.7	69.4 ± 10.7	< 0.001*
Male, n (%)	2895 (66.0)	2422 (67.3)	473 (60.1)	< 0.001*
Diabetes, n (%)	1058 (24.1)	836 (23.2)	222 (28.2)	0.004*
Prior PCI, n (%)	1088 (24.8)	920 (25.6)	168 (21.3)	0.013*
Prior MI, n (%)	340 (7.8)	283 (7.9)	57 (7.2)	0.555
LVEF, %	59.7 ± 13.0	60.4 ± 12.9	56.6 ± 13.3	< 0.001*
Average SBP, mmHg	123.2 ± 14.4	123.8 ± 14.1	120.6 ± 15.5	< 0.001*
**Nutritional status**				
NRS-2002 score	1.0 [0.0, 1.0]	1.0 [0.0, 1.0]	1.0 [1.0, 2.0]	< 0.001*
CONUT score	3.0 [1.0, 4.0]	3.0 [1.0, 4.0]	3.0 [2.0, 5.0]	< 0.001*
PNI score	45.8 [41.7, 49.7]	46.32 [42.5, 50.1]	43.5 [38.0, 48.2]	< 0.001*
GNRI score	98.6 [92.6, 103.3]	99.0 [93.5, 103.6]	95.9 [88.4, 101.7]	< 0.001*
**Laboratory data**				
Scr on admission, μmol/L	76.0 [64.0, 94.0]	76.0 [65.0, 93.0]	73.0 [60.0, 100.0]	0.090*
Scr elevation, %	5.2 [−3.9, 18.2]	1.9 [−5.7, 10.1]	43.2 [32.4, 68.1]	< 0.001*
eGFR, ml/min/1.73m^2^	78.7 ± 23.3	79.4 ± 22.1	75.6 ± 28.0	0.012*
Total cholesterol, mmol/L	4.09 ± 1.19	4.12 ± 1.18	3.99 ± 1.21	0.006*
Low density lipoprotein, mmol/L	2.22 ± 0.91	2.22 ± 0.91	2.20 ± 0.89	0.485
C-reactive protein, mg/L	2.3 [0.9, 8.0]	2.0 [0.8, 6.6]	4.4 [1.5, 16.0]	< 0.001*
Hemoglobin, g/L	128.1 ± 19.9	129.8 ± 18.9	120.6 ± 22.1	< 0.001*
Lymphocyte, × 10^9^/L	1.39 ± 0.60	1.41 ± 0.58	1.31 ± 0.67	< 0.001*
Serum albumin, g/L	38.6 ± 5.0	39.1 ± 4.7	36.6 ± 5.7	< 0.001*
**CAG/PCI data**				
CAG with PCI, n (%)	1993 (45.4)	1626 (45.2)	367 (46.6)	0.482
Volume of contrast agent, mg	80.0 [50.0, 130.0]	80.0 [50.0, 130.0]	80.0 [52.0, 140.0]	0.249
Type of contrast agent, n (%)				0.992
Isotonic	1387 (31.6)	1138 (31.6)	249 (31.6)	
Hypotonic	2999 (68.4)	2461 (68.4)	538 (68.4)	
Chronic total occlusion, n (%)	711 (16.2)	579 (16.1)	132 (16.8)	0.637
Total length of stents, mm	38.0 [25.0, 63.0]	39.0 [25.0, 64.0]	36.0 [24.0, 57.0]	0.101
**Pre-procedure medication, n (%)**				
Statin	3655 (83.3)	3052 (84.8)	603 (76.6)	< 0.001*
Aspirin	3632 (82.8)	3061 (85.1)	571 (72.6)	< 0.001*
Oral furosemide	1266 (28.9)	899 (25.0)	367 (46.6)	< 0.001*
Furosemide injection	660 (15.0)	421 (11.7)	239 (30.4)	< 0.001*
Dopamine	1231 (28.1)	929 (25.8)	302 (38.4)	< 0.001*

Data are mean ± standard deviation, median [interquartile range], or n (%). PCI indicates percutaneous coronary intervention; MI, myocardial infarction; LVEF, left ventricular ejection fraction; SBP, systolic blood pressure; NRS-2002, nutritional risk screening 2002; CONUT, controlling nutritional status; PNI, prognostic nutritional index; GNRI, geriatric nutritional risk index; Scr, serum creatinine; eGFR, estimated glomerular filtration rate; CAG, coronary angiography; CI-AKI, contrast-induced acute kidney injury. *P < 0.05

### The analysis of correlation

The correlation analysis was performed by using Spearman rank-order correlation. In Fig. 1B, the correlation matrix plot indicated the remarkable correlations between CONUT and PNI (ρ = − 0.74, *P* < 0.001), CONUT and GNRI (ρ = − 0.48, *P* < 0.001), GNRI and PNI (ρ = 0.80, *P* < 0.001), respectively. The proportion of Scr elevation was found to be positively correlated with NRS-2002 (ρ = 0.11, *P* < 0.001) and CONUT (ρ = 0.16, *P* < 0.001), while negatively correlated with PNI (ρ = − 0.19, *P* < 0.001) and GNRI (ρ = − 0.20, *P* < 0.001).

### The association between nutritional risk and the proportion of Scr elevation

The scatter plot with linear fits visualized the association between nutritional scores and the proportion of Scr elevation (Fig. S2). Moreover, multivariable linear regression analyses were performed and adjusted underlying confounders for CI-AKI. The results found that scores of NRS-2002 and CONUT linearly increased the proportion of Scr elevation (NRS-2002: β = 5.900, 95%CI [4.427 to 7.372], *P* < 0.001; CONUT: β = 2.239, 95%CI [1.419 to 3.060], *P* < 0.001), while scores of PNI and GNRI linearly decreased the proportion of Scr elevation (PNI: β = − 0.668, 95%CI [− 0.964 to − 0.373], *P* < 0.001; GNRI (β = − 0.568, 95%CI [− 0.808 to − 0.328], *P* < 0.001) (Table 2). These results suggest that nutritional risk is associated with a greater proportion of Scr elevation. The complete multivariable linear regression model (including confounders) is presented in Table S2–3.

**Table 2:** Association between nutritional scores and the proportion of serum creatinine elevation by linear regression analyses

	Model 1 (Crude)		Model 2 (Adjusted)		Model 3 (Adjusted)	
	β [95% CI]	*P* value	β [95% CI]	*P* value	β [95% CI]	*P* value
NRS-2002	6.338 [5.174 to 7.501]	< 0.001	6.234 [4.748 to 7.720]	< 0.001	5.900 [4.427 to 7.372]	< 0.001
CONUT	3.687 [3.028 to 4.347]	< 0.001	2.690 [1.863 to 3.518]	< 0.001	2.239 [1.419 to 3.060]	< 0.001
PNI	−1.269 [−1.493 to −1.045]	< 0.001	−0.882 [−1.178 to −0.586]	< 0.001	−0.668 [−0.964 to −0.373]	< 0.001
GNRI	−1.002 [− 1.198 to − 0.805]	< 0.001	−0.734 [− 0.975 to − 0.493]	< 0.001	−0.568 [− 0.808 to − 0.328]	< 0.001

Model 1 adjusted for none

Model 2 adjusted for age (except NRS-2002), gender, diabetes, average SBP, eGFR, LVEF, hemoglobin, C-reactive protein, the volume of contrast agent consumption, the type of contrast agent

Model 3 additionally adjusted for pre-procedure medication, including statin, furosemide, and dopamine

The NRS-2002 scores already took age into account, and thus age was not adjusted in the multivariable model. The complete multivariable linear regression model (including covariates) is presented in Table S2–3. CI indicates confidence interval. Other abbreviations refer to Table 1

### The association between nutritional risk and CI-AKI

Subjects were divided into four categories based on the nutritional score distribution. The category with the lowest nutritional risk was set as a reference (NRS-2002, 0; CONUT, 0–1; PNI, ≥52; GNRI, ≥104). In Table 3, multivariable logistic regression analyses indicated that nutritional risk was significantly associated with CI-AKI. This result was consistent when four different nutritional tools were employed, including NRS-2002 ([3–7 vs. 0]: OR = 4.026, 95%CI [2.732 to 5.932], *P* < 0.001), CONUT ([6–12 vs. 0–1]: OR = 2.230, 95%CI [1.586 to 3.136], *P* < 0.001), PNI ([< 38 vs. ≥52]: OR = 2.349, 95%CI [1.529 to 3.610], *P* < 0.001), and GNRI ([< 90 vs. ≥104]: OR = 1.822, 95%CI [1.229 to 2.702], *P* = 0.003) (Table 3). The linear trend of coefficients was also identified among consecutive categories (all *P* values ≤0.001). The complete multivariable logistic regression model (including confounders) is presented in Table S4–5.

**Table 3:** Association between nutritional scores and CI-AKI by logistic regression analyses

Nutrition indicators	Score	Model 1 (Crude)		Model 2 (Adjusted)		Model 3 (Adjusted)	
		Odds ratio [95% CI]	*P* value	Odds ratio [95% CI]	*P* value	Odds ratio [95% CI]	*P* value
NRS-2002	0	1 (reference)		1 (reference)		1 (reference)	
	1	1.580 [1.276 to 1.956]	< 0.001*	1.330 [1.045 to 1.694]	0.021*	1.364 [1.067 to 1.744]	0.013*
	2	2.437 [1.880 to 3.159]	< 0.001*	1.811 [1.341 to 2.447]	< 0.001*	1.790 [1.319 to 2.430]	< 0.001*
	3–7	5.796 [4.278 to 7.854]	< 0.001*	3.915 [2.678 to 5.723]	< 0.001*	4.026 [2.732 to 5.932]	< 0.001*
	*P* for trend		< 0.001*		< 0.001*		< 0.001*
CONUT	0–1	1 (reference)		1 (reference)		1 (reference)	
	2–3	1.040 [0.816 to 1.325]	0.754	1.043 [0.796 to 1.367]	0.762	1.059 [0.806 to 1.392]	0.682
	4–5	1.617 [1.248 to 2.095]	< 0.001*	1.401 [1.039 to 1.890]	0.027*	1.308 [0.964 to 1.773]	0.084
	6–12	3.470 [2.625 to 4.587]	< 0.001*	2.473 [1.771 to 3.451]	< 0.001*	2.230 [1.586 to 3.136]	< 0.001*
	*P* for trend		< 0.001*		< 0.001*		< 0.001*
PNI	< 38	4.516 [3.200 to 6.373]	< 0.001*	2.798 [1.841 to 4.253]	< 0.001*	2.349 [1.529 to 3.610]	< 0.001*
	38–44	1.664 [1.213 to 2.282]	0.002*	1.192 [0.820 to 1.733]	0.356	1.068 [0.730 to 1.561]	0.735
	45–51	1.174 [0.857 to 1.609]	0.317	1.002 [0.700 to 1.434]	0.991	0.960 [0.668 to 1.379]	0.825
	≥52	1 (reference)		1 (reference)		1 (reference)	
	*P* for trend		< 0.001*		< 0.001*		< 0.001*
GNRI	< 90	3.203 [2.327 to 4.408]	< 0.001*	2.122 [1.447 to 3.112]	< 0.001*	1.822 [1.229 to 2.702]	0.003*
	90–96	1.321 [0.954 to 1.831]	0.094	1.097 [0.758 to 1.588]	0.622	1.009 [0.693 to 1.469]	0.961
	97–103	1.196 [0.879 to 1.627]	0.255	1.081 [0.764 to 1.529]	0.660	1.035 [0.728 to 1.469]	0.849
	≥104	1 (reference)		1 (reference)		1 (reference)	
	*P* for trend		< 0.001*		< 0.001*		0.001*

Model 1 adjusted for none

Model 2 adjusted for age (per 10 years, except NRS-2002), gender (male or female), diabetes (yes or no), average SBP (< 90, 90–114, 115–139, ≥140 mmHg), eGFR (< 30, 30–59, 60–89, ≥90 ml/min/1.73m^2^), LVEF (< 50, 50–64, ≥65%), hemoglobin (< 110, 110–139, ≥140 g/L), C-reactive protein (< 5, 5–10, ≥10 mg/L), the volume of contrast agent consumption (< 60, 60–119, ≥120 mg), and the type of contrast agent (isotonic or hypotonic)

Model 3 additionally adjusted for pre-procedure medication, including statin (yes or no), furosemide (yes or no), and dopamine (yes or no)

We performed tests for the linear trend of coefficients by entering the median value of each category as a continuous variable in the models. The NRS-2002 scores already took age into account, and thus age was not adjusted in the multivariable model. The complete multivariable logistic regression model (including covariates) is presented in Table S4–5. CI indicates confidence interval. Other abbreviations refer to Table 1. *P < 0.05

**Fig. 2:**
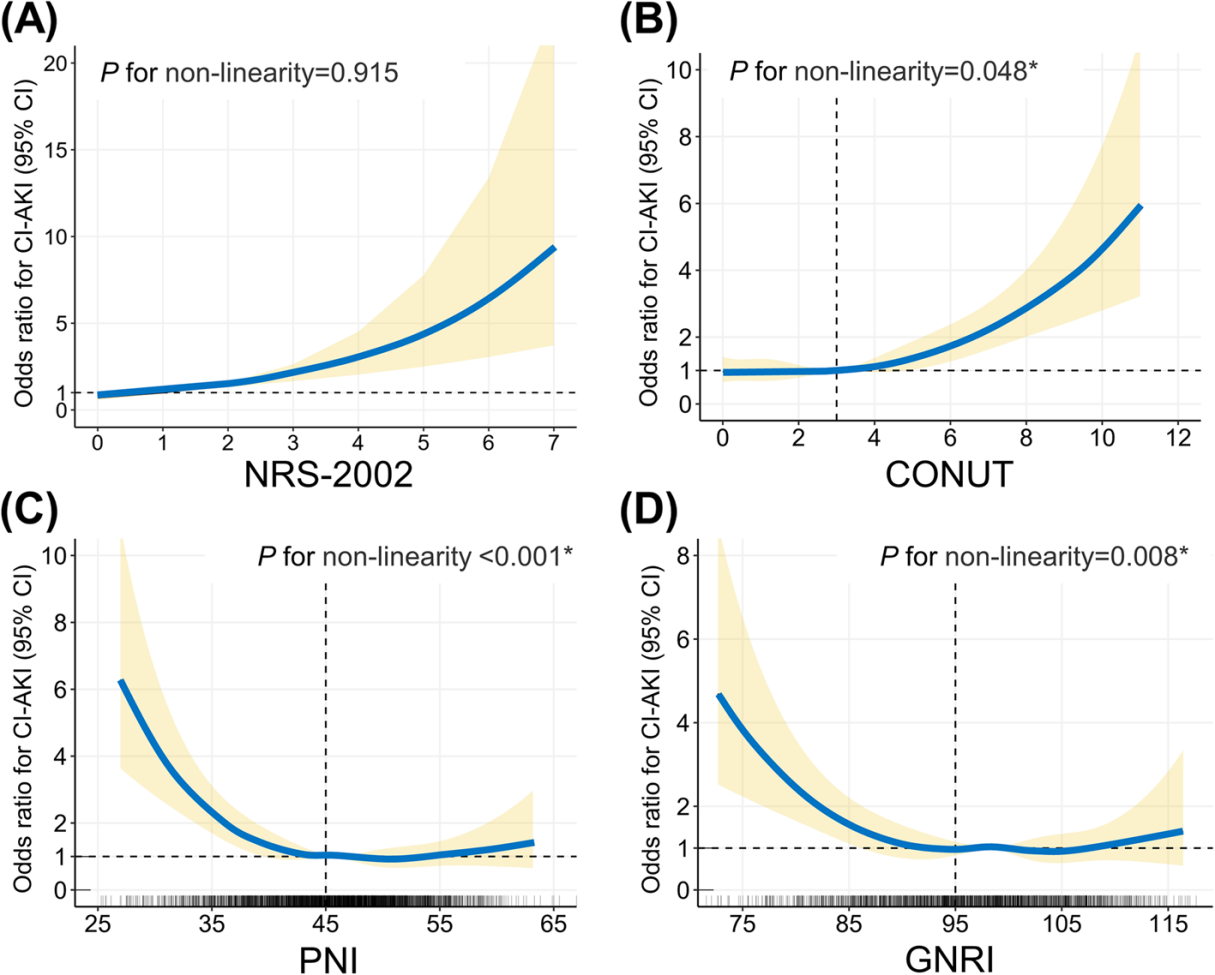
Restricted cubic spline analyses between nutritional risk and CI-AKI. The restricted cubic spline plot visualizes the association between CI-AKI and nutritional scores, including (**A**) NRS-2002, (**B**) CONUT, (**C**) PNI, and (**D**) GNRI. The spline model adjusted for underlying clinical confounders, including age (except NRS-2002), gender, diabetes, average SBP, eGFR, LVEF, hemoglobin, C-reactive protein, the volume of contrast agent consumption, the type of contrast agent, pre-procedure medications (statin, furosemide, and dopamine). Abbreviations refer to Fig. 1. *P < 0.05

In Fig. 2, restricted cubic spline models visualize the association between nutritional scores and CI-AKI risks. NRS-2002 tends to increase the risk of CI-AKI by a linear trend (*P* for non-linearity = 0.915, Fig. 2A). For CONUT, the spline model indicates a relatively flat curve until CONUT is around 3 and then CI-AKI risks start to increase rapidly afterward (*P* for non-linearity = 0.048, Fig. 2B). For PNI and GNRI, CI-AKI risks rapidly decrease until around 45 for PNI (Fig. 2C) and 95 for GNRI (Fig. 2D), and then the curve turns flat (*P* for non-linearity: PNI, < 0.001; GNRI, 0.008).

### The ROC analyses and subgroup analyses

**Fig. 3:**
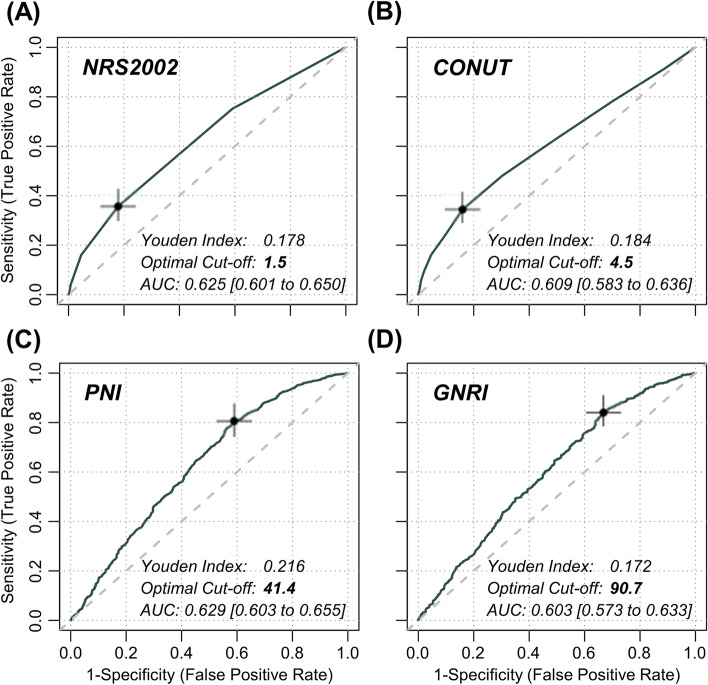
Receiver operating characteristic (ROC) analyses between nutritional scores and CI-AKI The ROC curves depict the predictive performance of (**A**) NRS-2002, (**B**) CONUT, (**C**) PNI, and (**D**) GNRI on CI-AKI, respectively. The maximum value of the Youden index determines the optimal cut-off point for CI-AKI and is marked with a cross in the plot. The AUC was calculated for each nutritional scores. AUC indicates area under the curve; other abbreviations, refer to Fig. 1

ROC analyses of nutritional scores on CI-AKI are presented in Fig. 3. Nutritional scores showed the excellent prediction performance to CI-AKI with the area under the curve (AUC) being presented, including NRS-2002 (AUC = 0.625, 95%CI [0.601 to 0.650]), CONUT (AUC = 0.609, 95%CI [0.583 to 0.636]), PNI (AUC = 0.629, 95%CI [0.603 to 0.655]), and GNRI (AUC = 0.603, 95%CI [0.573 to 0.633]). According to the maximum value of the Youden index, optimal cut-off points for CI-AKI were determined to be 1.5 for NRS-2002, 4.5 for CONUT, 41.4 for PNI, and 90.7 for GNRI.

**Fig. 4:**
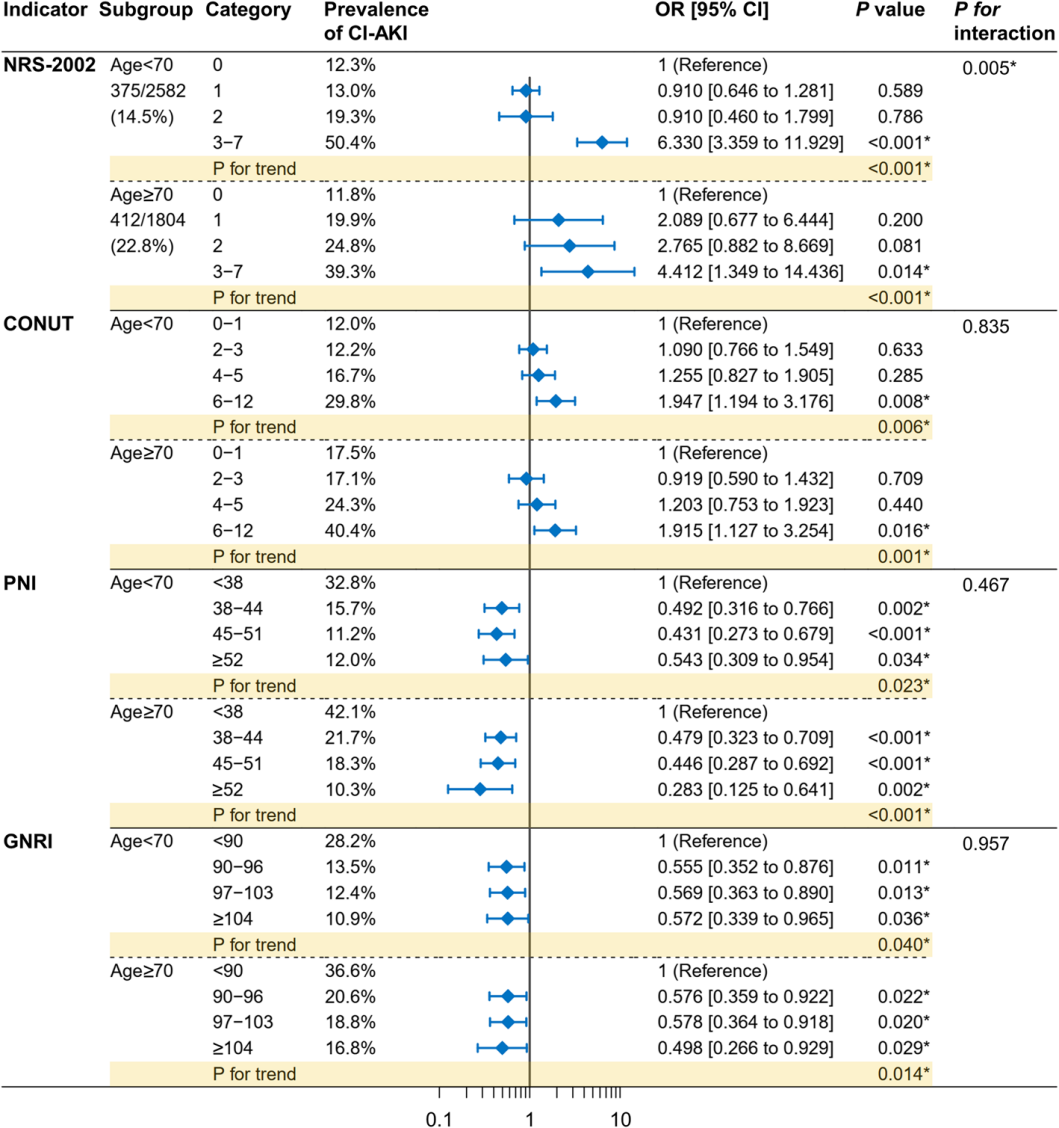
Subgroup analyses according to age. Patients were divided into groups according to the age (< 70 or ≥ 70 years). Multivariable logistic regression analyses were performed. The category with the lowest nutritional score was set to be the reference. *P* for trend was calculated by entering the median value of each category as a continuous variable in the models. Tests for interaction (nutritional categories × subgroup stratification) were performed by the likelihood ratio test. Abbreviations refer to Fig. 1. *P < 0.05

Subgroup analyses with interaction testing were performed according to age (< 70/≥70 years, Fig. 4), gender (male/female, Fig. S3), PCI (with/without, Fig. S4), and eGFR (< 60/≥60 ml/min/1.73m^2^, Fig. S5). The association between nutritional risk and CI-AKI was consistent when subgroup analyses were performed. The only statistically significant interaction effect was identified between NRS-2002 and age stratification (*P* for interaction = 0.005).

## Discussion

In this retrospective cross-sectional study, a total of 4386 patients undergoing CAG or PCI were enrolled. Nutritional risk was identified as a potential risk factor for CI-AKI. High scores of NRS-2002 and CONUT, low scores of PNI and GNRI were found to be associated with an increased incidence of CI-AKI. These findings were consistent across subgroups stratified by age, gender, PCI, and eGFR. Moreover, a good predictive performance of the nutrition scores for CI-AKI was also identified along with optimal cut-points being determined.

Nutritional risk is prevalent in numerous age-related degenerative diseases, and its presence often indicates a poor clinical prognosis [17]. Nutrition screening tools provide a comprehensive and objective assessment of nutritional status by integrating various patient-level parameters, such as albumin levels, lymphocyte counts, and body mass index. In this study, four nutritional screening tools (NRS-2002, CONUT, PNI, and GNRI) were employed to assess the nutritional risk of patients. These nutritional tools are widely used in clinical practice and have been found to be associated with many diseases. By using nutritional screening tools (CONUT, PNI, and nutritional risk index), Roubín et al. reported that approximately 10–40% of acute coronary syndrome patients had moderate to severe nutritional risk [18]. More importantly, the presence of nutritional risk increases the incidence of subsequent adverse cardiovascular events [18]. By using NRS-2002, Li et al. found that nutritional risk (NRS-2002 ≥ 3) increased the incidence of acute kidney injury in hospitalized patients and contributed to a poor clinical prognosis [29]. Moreover, by using GNRI, Liu et al. reported that on-admission nutritional risk was a potential risk factor for the subsequent mortality in elderly patients with intensive care [30]. Consistently, the current study demonstrated that nutritional risk is associated with a higher incidence of CI-AKI in patients undergoing CAG or PCI. Furthermore, this finding was verified by using four different nutritional screening tools.

Several potential pathological mechanisms may account for our findings. First, nutritional risks may increase the incidence of CI-AKI by suppressing bone marrow hematopoiesis. Bone marrow is deemed to be one of the most important hematopoietic organs. The presence of nutritional risk suggests an underlying malnutrition status, which can disturb the microenvironment of the bone marrow stroma and impair hematopoiesis, thereby reducing the synthesis of hemoglobin and erythrocyte [31]. A deficiency in hemoglobin and erythrocyte decreases the oxygen supply to the kidney, thereby increasing the incidence of CI-AKI [32]. Second, nutritional risks may increase the incidence of CI-AKI by upregulating inflammation levels. Malnutrition has been shown to be associated with the upregulation of inflammation levels in patients with renal dysfunction [33]. While inflammation upregulation is also a major risk factor for CI-AKI [34]. As in the current study, CI-AKI patients had a higher CRP level (CI-AKI vs. non-CI-AKI: 4.4 [1.5, 16.0] vs. 2.0 [0.8, 6.6] mg/L, *P* < 0.001). Elevated CRP downregulates endothelial nitric oxide synthase activity and inhibits nitric oxide production [35]. Nitric oxide has multiple protective effects on the regulation of the cardiovascular and renal systems, and its reduction may disrupt the regulation of renal vessels, thereby increasing the incidence of CI-AKI [36]. Third, nutritional risks may increase the incidence of CI-AKI by promoting dysregulated immune surveillance. Malnutrition has been shown to disturb the metabolism and function of immune cells and promote immunosuppression [37]. The resident and infiltrating phagocytes of the kidney may also be affected by malnutrition. Dysfunctional immune surveillance of renal phagocytes has been shown to be one of the pathological mechanisms of CI-AKI [38].

Despite the four different nutritional screening tools being used, the relationship between nutritional risk and CI-AKI seems to be consistent. This relationship may be intrinsic and independent of the nutritional tool itself. Moreover, the current study assessed the interaction between nutrition categories and stratified factors, including age (< 70/≥70 years), gender (male/female), PCI (with/without), and eGFR (< 60/≥60 ml/min/1.73m^2^). Most of the interaction tests were not significant, indicating our findings were consistent across different subgroups. However, a significant interaction between NRS-2002 categories and age stratification (< 70/≥70 years) was identified (P for interaction = 0.005). NRS-2002 assigns a score for patients older than 70, which may account for this interaction [24]. In younger patients (< 70 years), the risk of CI-AKI appears to increase significantly only when the NRS-2002 score is ≥3; whereas, in older patients (≥70 years), this risk appears to increase gradually.

Despite the important findings being mentioned, some limitations need to be recognized. First, this is a retrospective study, in which inherent bias exists. Second, the cross-sectional design prohibits causal interpretations of the association between nutritional risk and CA-AKI. Third, this study has limited generalizability as all the patients enrolled in this study were Chinese. Fourth, four different nutritional screening tools were pre-determined according to the data available in this retrospective study. Therefore, the selection bias of the nutritional tools cannot be avoided. Fifth, the nutritional risk was assessed on admission. The subsequent potential nutritional treatment was not considered, which may affect nutrition scores.

## Conclusion

In patients undergoing CAG, nutritional risks (high scores of NRS-2002 and CONUT; low scores of PNI and GNRI) were associated with CI-AKI. Pre-procedural nutritional interventions may be helpful in reducing the incidence of CI-AKI.

## Supplementary Information

**Additional file 1.**

## Data Availability

The datasets used and/or analyzed during the current study are available from the corresponding author on reasonable request.

## Abbreviations

CAD: Coronary artery disease
CAG: Coronary angiography
PCI: pPercutaneous coronary intervention
CI-AKI: Contrast-induced acute kidney injury
Scr: Serum creatinine
NRS-2002: Nutritional risk screening 2002
CONUT: Controlling nutritional status
PNI: Prognostic nutritional index
GNRI: Geriatric nutritional risk index
eGFR: estimated glomerular filtration rate
ROC: Receiver operating characteristic
AUC: Area under the curve
