# Supplementary material for: The association between nutritional risk and contrast-induced acute kidney injury in patients undergoing coronary angiography: a cross-sectional study
Source: Nutr J. 2022 Sep 16;21:56. doi: 10.1186/s12937-022-00810-z (PMC9479352; doi:10.1186/s12937-022-00810-z)
Supplement: Supplementary file 1 — Additional file 1. [file 12937_2022_810_MOESM1_ESM.docx]

**Supplementary Material**

**Supplementary Figures**

**Figure S1.** Flow chart of the current study

**Figure S2.** Scatter plot with linear fits between nutritional scores and the proportion of Scr elevation

**Figure S3.** Subgroup analysis according to gender (male or female)

**Figure S4.** Subgroup analysis according to PCI (CAG / CAG with PCI)

**Figure S5.** Subgroup analysis according to eGFR (<60 or ≥60 ml/min/1.73m^2^)

**Supplementary Tables**

**Table S1.** Scoring system for the CONUT

**Table S2.** Multivariable linear regression models on Scr elevation

**Table S3.** Multivariable linear regression models on Scr elevation (with additional adjustment for medications)

**Table S4.** Multivariable logistic regression models on CI-AKI

**Table S5.** Multivariable logistic regression models on CI-AKI (with additional adjustment for medications)

**Figure S1.** Flow chart of the current study


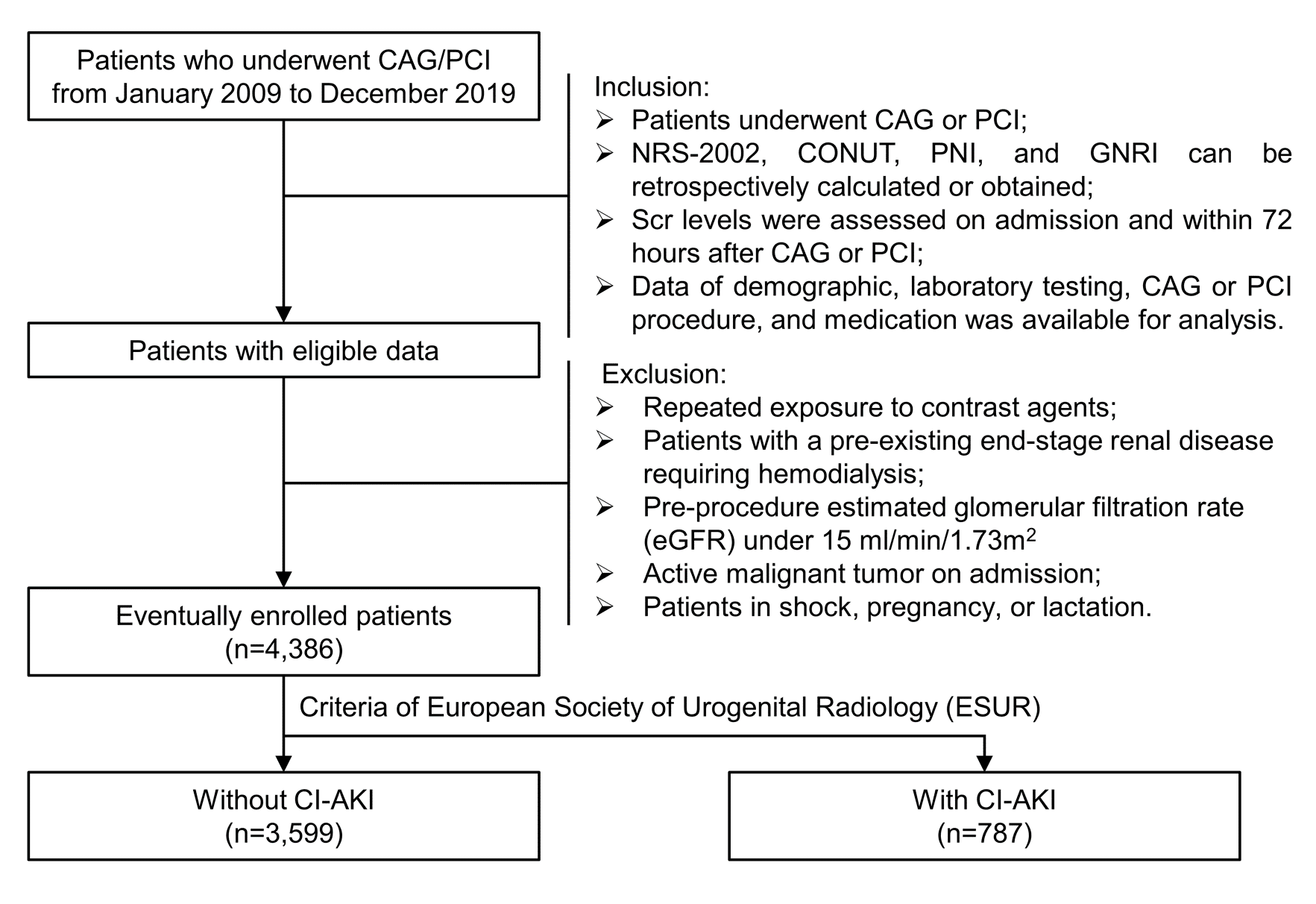


CAG indicates coronary angiography; PCI, percutaneous coronary intervention; NRS-2002, nutritional risk screening 2002; CONUT, controlling nutritional status; PNI, prognostic nutritional index; GNRI, geriatric nutritional risk index; Scr, serum creatinine; eGFR, estimated glomerular filtration rate; CI-AKI, contrast-induced acute kidney injury.

**Figure S2.** Scatter plot with linear fits between nutritional scores and the proportion of Scr elevation


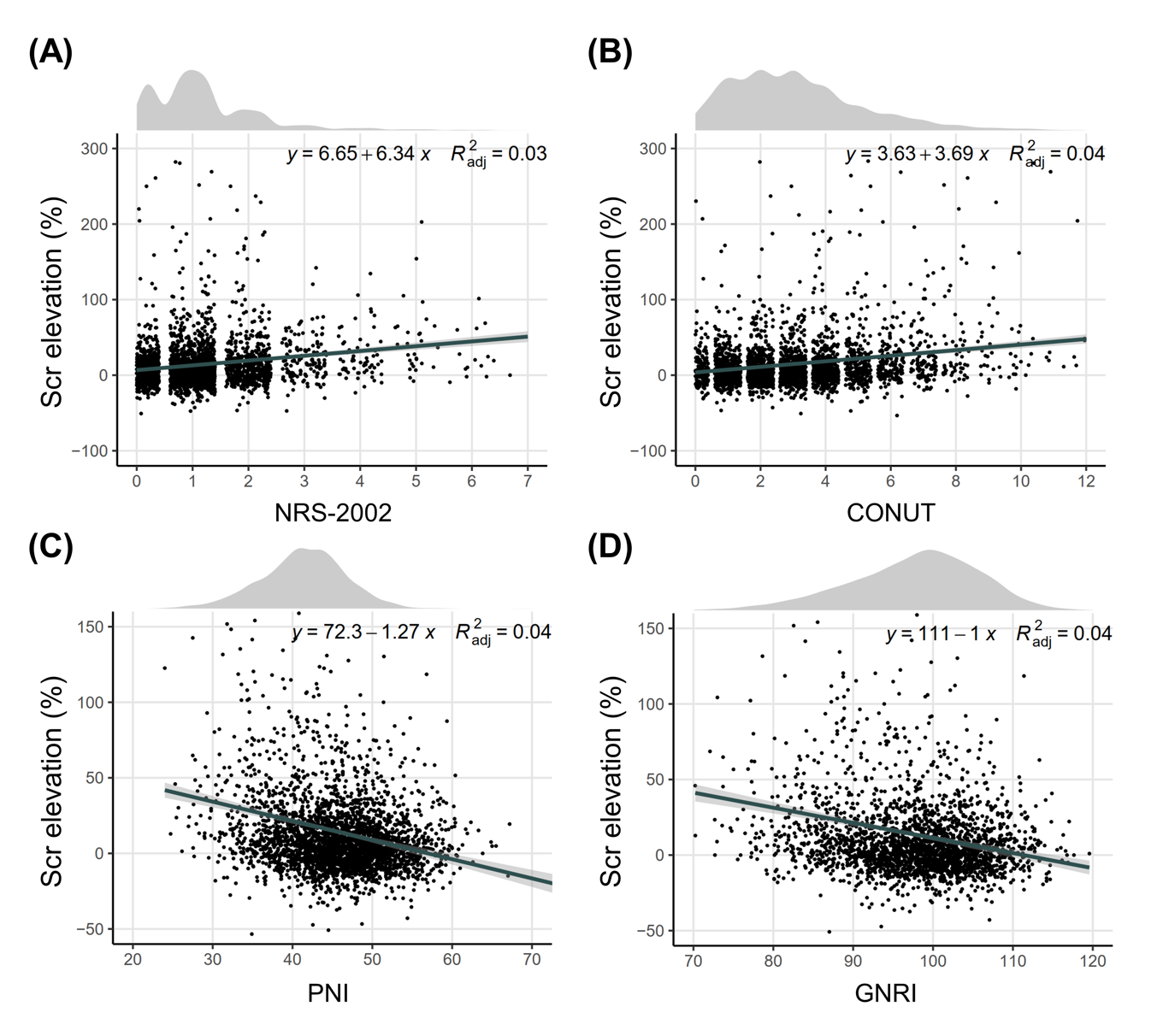


The association between Scr elevation and nutritional scores was visualized by scatter plots with linear fits. Nutritional scores included (A) NRS-2002, (B) CONUT, (C) PNI, and (D) GNRI. Regression equations, adjusted R^2^ and density plot of nutritional scores were shown in the diagram. Abbreviations refer to Figure S1.

**Figure S3.** Subgroup analysis according to the gender (male or female)


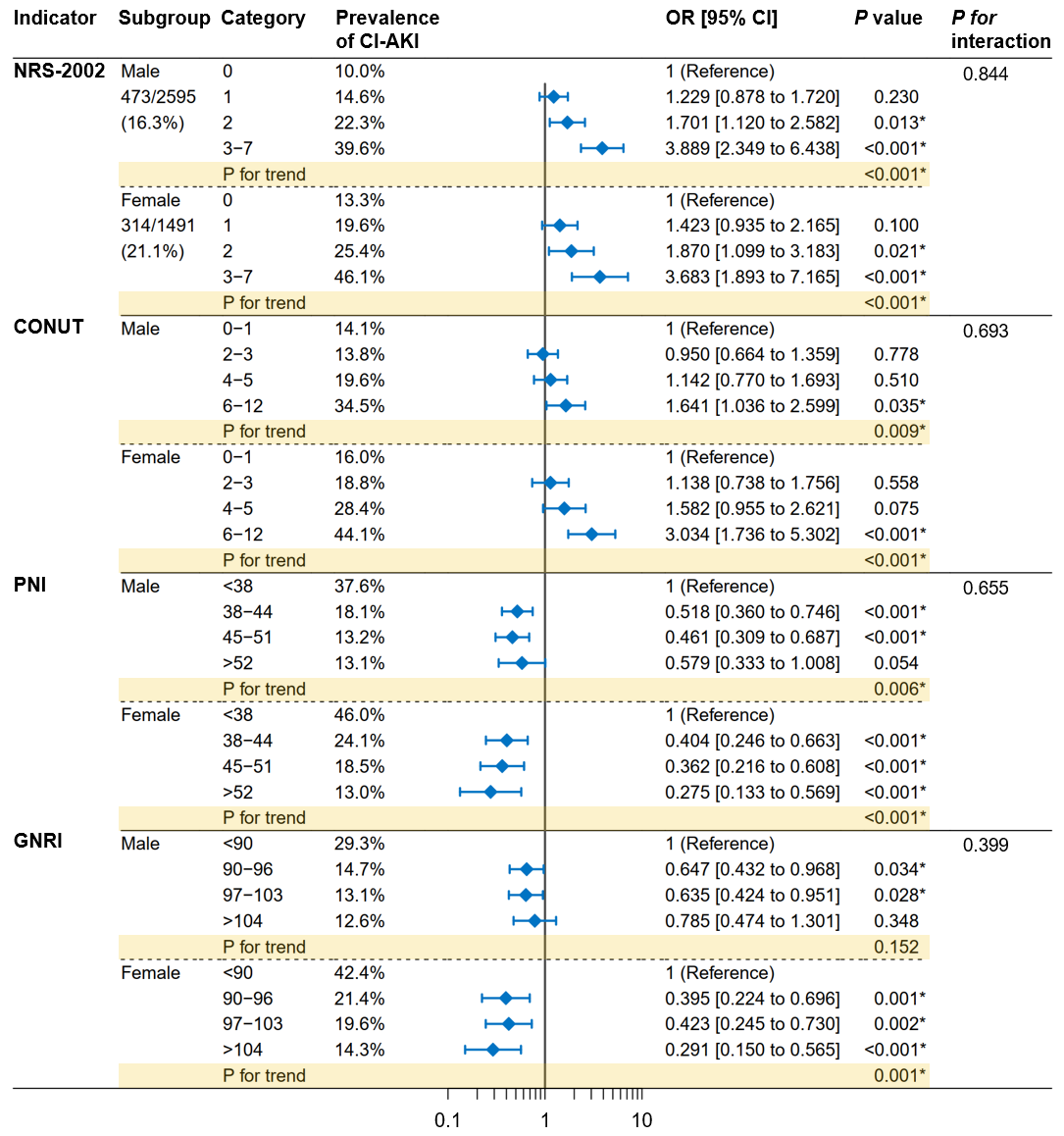


Patients were divided into groups according to the gender (male or female). Multivariable logistic regression analyses were performed by adjusting underlying confounders, including age (except NRS-2002), diabetes, average SBP, LVEF, hemoglobin, C-reactive protein, eGFR, the volume of contrast agent consumption, the type of contrast agent, pre-procedure medications (statin, furosemide, and dopamine).. The category with the lowest nutritional score was set to be the reference. P for trend was calculated by entering the median value of each category as a continuous variable in the models. Tests for interaction (nutritional categories × subgroup stratification) were performed with the likelihood ratio test. Abbreviations refer to Figure 1. *P <0.05

**Figure S4.** Subgroup analysis according to the PCI (CAG / CAG with PCI)


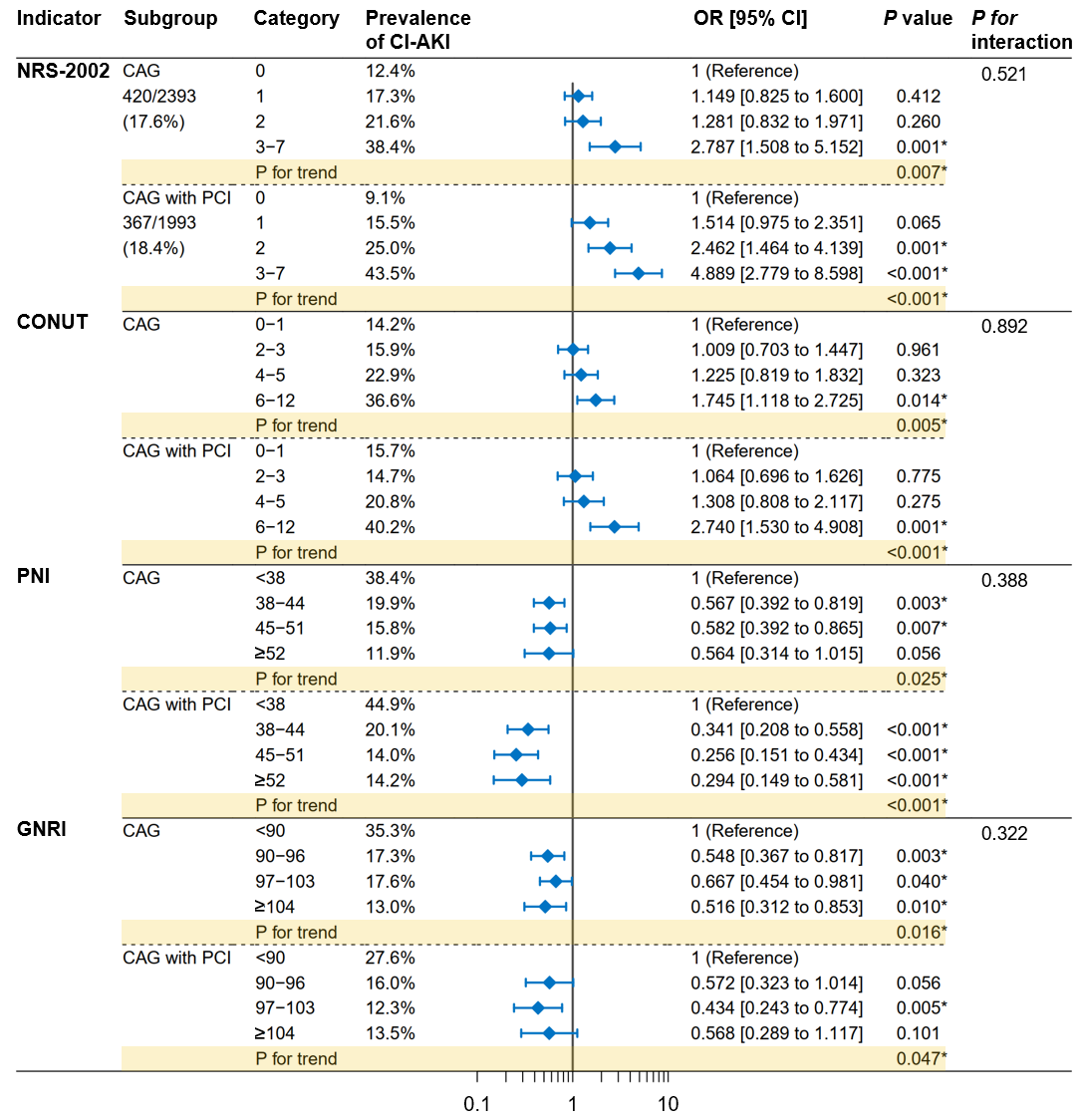


Patients were grouped according to whether they underwent PCI (CAG / CAG with PCI). Multivariable logistic regression analyses were performed by adjusting underlying confounders, including age (except NRS-2002), gender, diabetes, average SBP, LVEF, hemoglobin, C-reactive protein, eGFR, the volume of contrast agent consumption, the type of contrast agent, pre-procedure medications (statin, furosemide, and dopamine).. The category with the lowest nutritional score was set to be the reference. P for trend was calculated by entering the median value of each category as a continuous variable in the models. Tests for interaction (nutritional categories × subgroup stratification) were performed with the likelihood ratio test. Abbreviations refer to Figure 1. *P <0.05

**Figure S5.** Subgroup analysis according to the eGFR (<60 or ≥60 ml/min/1.73m^2^)


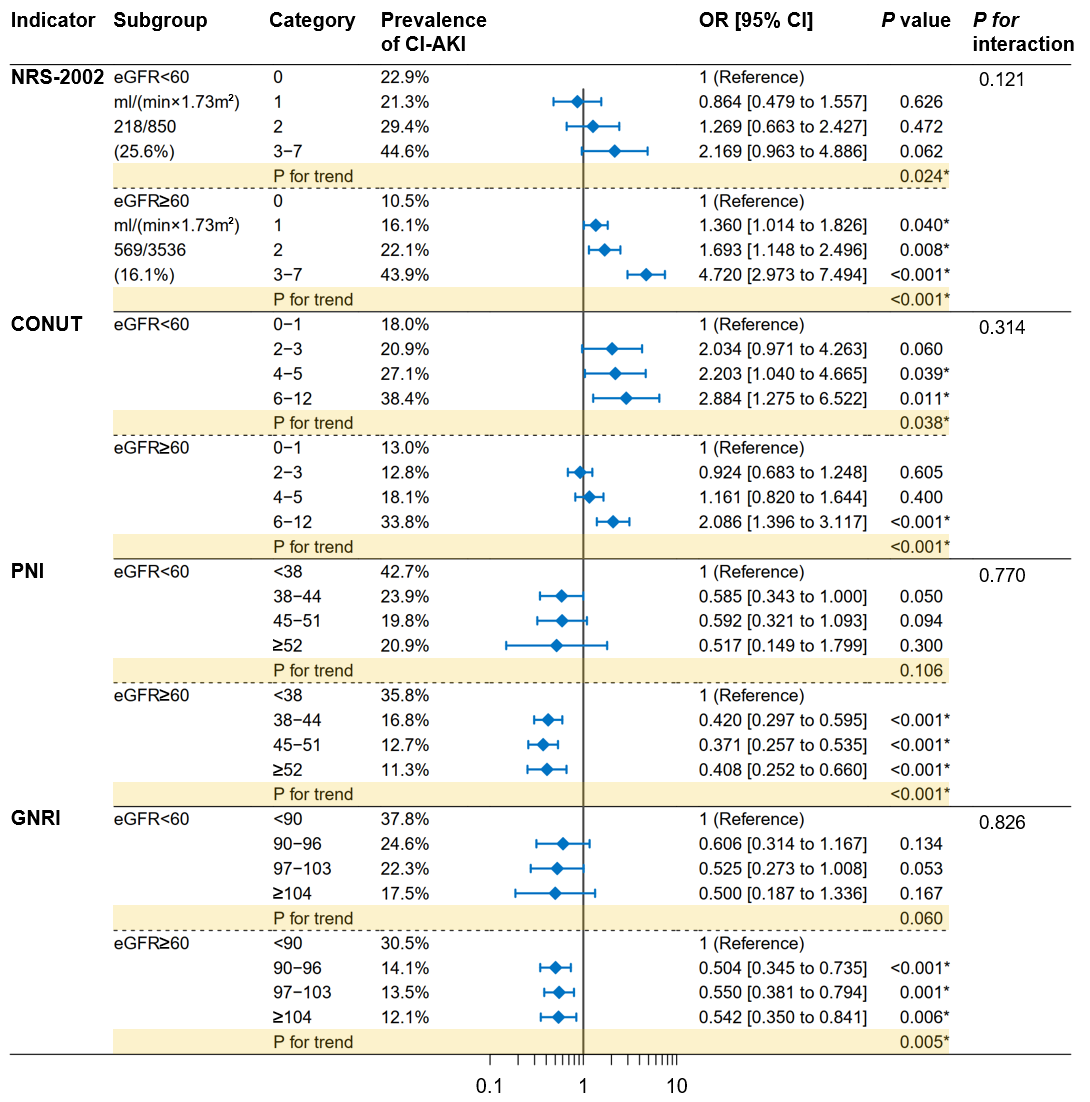


Patients were divided into groups according to the eGFR (<60 or ≥60 ml/min/1.73m^2^). Multivariable logistic regression analyses were performed by adjusting underlying confounders, including age (except NRS-2002), gender, diabetes, average SBP, LVEF, hemoglobin, C-reactive protein, the volume of contrast agent consumption, the type of contrast agent, pre-procedure medications (statin, furosemide, and dopamine). The category with the lowest nutritional score was set to be the reference. P for trend was calculated by entering the median value of each category as a continuous variable in the models. Tests for interaction (nutritional categories × subgroup stratification) were performed with the likelihood ratio test. Abbreviations refer to Figure 1. *P <0.05

**Table S1.** Scoring system for the CONUT

| Parameter | None | Light | Moderate | Severe |
| --- | --- | --- | --- | --- |
| Serum albumin (g/dL) | ≥ 3.50 | 3.00-3.49 | 2.50-2.99 | < 2.50 |
| Score | 0 | 2 | 4 | 6 |
| Total lymphocyte count (/mm^3^) | ≥ 1600 | 1200-1599 | 800-1199 | < 800 |
| Score | 0 | 1 | 2 | 3 |
| Total cholesterol (mg/dL) | ≥ 180 | 140-179 | 100-139 | < 100 |
| Score | 0 | 1 | 2 | 3 |

Abbreviations refer to Table 1.

**Table S2.** Multivariable linear regression models on Scr elevation

|  | NRS-2002 |  |  | CONUT |  |  | PNI |  |  | GNRI |  |
| --- | --- | --- | --- | --- | --- | --- | --- | --- | --- | --- | --- |
|  | Coefficient β | *P* value |  | Coefficient β | *P* value |  | Coefficient β | *P* value |  | Coefficient β | *P* value |
| Nutritional score | 6.234 [4.748 to 7.72] | <0.001* |  | 2.690 [1.863 to 3.518] | <0.001* |  | -0.882 [-1.178 to -0.586] | <0.001* |  | -0.734 [-0.975 to -0.493] | <0.001* |
| Age, per 10 years | - | - |  | 0.683 [-0.912 to 2.279] | 0.401 |  | 0.556 [-1.051 to 2.163] | 0.497 |  | 0.687 [-1.128 to 2.502] | 0.458 |
| Male | 1.727 [-1.276 to 4.730] | 0.260 |  | -0.324 [-3.863 to 3.215] | 0.858 |  | 0.140 [-3.386 to 3.666] | 0.938 |  | -1.560 [-5.523 to 2.404] | 0.440 |
| Diabetes | 1.722 [-1.524 to 4.968] | 0.299 |  | 0.344 [-3.367 to 4.056] | 0.856 |  | 0.840 [-2.875 to 4.556] | 0.658 |  | 0.763 [-3.404 to 4.929] | 0.720 |
| Average SBP, mmHg | -0.192 [-0.292 to -0.092] | <0.001* |  | -0.219 [-0.337 to -0.102] | <0.001* |  | -0.230 [-0.348 to -0.113] | <0.001* |  | -0.224 [-0.356 to -0.092] | 0.001* |
| eGFR, ml/min/1.73 m^2^ | 0.152 [0.084 to 0.220] | <0.001* |  | 0.177 [0.095 to 0.259] | <0.001* |  | 0.178 [0.096 to 0.260] | <0.001* |  | 0.118 [0.026 to 0.211] | 0.012* |
| Ejection fraction, % | -0.264 [-0.371 to -0.156] | <0.001* |  | -0.321 [-0.448 to -0.193] | <0.001* |  | -0.311 [-0.439 to -0.183] | <0.001* |  | -0.262 [-0.407 to -0.116] | <0.001* |
| Hemoglobin, g/L | -0.356 [-0.434 to -0.277] | <0.001* |  | -0.300 [-0.399 to -0.202] | <0.001* |  | -0.302 [-0.402 to -0.203] | <0.001* |  | -0.283 [-0.392 to -0.174] | <0.001* |
| C-reactive protein, mg/L | 0.148 [0.086 to 0.209] | <0.001* |  | 0.091 [0.023 to 0.159] | 0.009* |  | 0.084 [0.015 to 0.153] | 0.017* |  | 0.105 [0.020 to 0.191] | 0.016* |
| Volume of contrast agent, mg | -0.023 [-0.042 to -0.004] | 0.015* |  | -0.022 [-0.045 to 0] | 0.054 |  | -0.022 [-0.045 to 0.001] | 0.059 |  | -0.029 [-0.054 to -0.004] | 0.025* |
| Isotonic agent (vs. hypotonic) | -0.599 [-3.634 to 2.437] | 0.699 |  | 0.223 [-3.483 to 3.930] | 0.906 |  | 0.317 [-3.392 to 4.027] | 0.867 |  | 0.490 [-3.715 to 4.695] | 0.819 |

The NRS-2002 scores already took age into account, and thus age was not adjusted in the multivariable model. Abbreviations refer to Table 1.

*P <0.05

**Table S3.** Multivariable linear regression models on Scr elevation (with additional adjustment for medications)

|  | NRS-2002 |  |  | CONUT |  |  | PNI |  |  | GNRI |  |
| --- | --- | --- | --- | --- | --- | --- | --- | --- | --- | --- | --- |
|  | Coefficient β | *P* value |  | Coefficient β | *P* value |  | Coefficient β | *P* value |  | Coefficient β | *P* value |
| Nutritional score | 5.900 [4.427 to 7.372] | <0.001* |  | 2.239 [1.419 to 3.060] | <0.001* |  | -0.668 [-0.964 to -0.373] | <0.001* |  | -0.568 [-0.808 to -0.328] | <0.001* |
| Age, per 10 years | - | - |  | 0.898 [-0.674 to 2.469] | 0.263 |  | 0.853 [-0.731 to 2.437] | 0.291 |  | 0.978 [-0.802 to 2.758] | 0.282 |
| Male | 1.885 [-1.075 to 4.844] | 0.212 |  | 0.246 [-3.241 to 3.733] | 0.890 |  | 0.765 [-2.711 to 4.242] | 0.666 |  | -0.485 [-4.378 to 3.408] | 0.807 |
| Diabetes | 1.595 [-1.612 to 4.803] | 0.330 |  | 0.526 [-3.138 to 4.190] | 0.778 |  | 0.921 [-2.748 to 4.589] | 0.623 |  | 1.304 [-2.788 to 5.396] | 0.532 |
| Average SBP, mmHg | -0.127 [-0.227 to -0.027] | 0.013* |  | -0.123 [-0.241 to -0.006] | 0.040* |  | -0.135 [-0.253 to -0.018] | 0.024* |  | -0.108 [-0.240 to 0.024] | 0.108 |
| eGFR, ml/min/1.73m^2^ | 0.165 [0.098 to 0.232] | <0.001* |  | 0.192 [0.111 to 0.273] | <0.001* |  | 0.193 [0.112 to 0.274] | <0.001* |  | 0.140 [0.050 to 0.231] | 0.002* |
| Ejection fraction, % | -0.067 [-0.181 to 0.048] | 0.253 |  | -0.124 [-0.257 to 0.010] | 0.070 |  | -0.117 [-0.251 to 0.017] | 0.086 |  | -0.071 [-0.220 to 0.078] | 0.350 |
| Hemoglobin, g/L | -0.317 [-0.394 to -0.239] | <0.001* |  | -0.264 [-0.361 to -0.167] | <0.001* |  | -0.275 [-0.373 to -0.177] | <0.001* |  | -0.243 [-0.349 to -0.136] | <0.001* |
| C-reactive protein, mg/L | 0.115 [0.054 to 0.176] | <0.001* |  | 0.056 [-0.011 to 0.124] | 0.103 |  | 0.053 [-0.015 to 0.121] | 0.127 |  | 0.071 [-0.013 to 0.156] | 0.096 |
| Volume of contrast agent, mg | -0.022 [-0.042 to -0.002] | 0.032* |  | -0.021 [-0.045 to 0.003] | 0.089 |  | -0.021 [-0.045 to 0.003] | 0.085 |  | -0.022 [-0.048 to 0.005] | 0.111 |
| Isotonic agent (vs. hypotonic) | -0.926 [-3.921 to 2.070] | 0.545 |  | -0.480 [-4.138 to 3.179] | 0.797 |  | -0.382 [-4.046 to 3.282] | 0.838 |  | -0.123 [-4.251 to 4.006] | 0.954 |
| Pre-procedure statin use | -11.035 [-14.743 to -7.327] | <0.001* |  | -10.937 [-15.047 to -6.828] | <0.001* |  | -10.854 [-14.989 to -6.718] | <0.001* |  | -12.081 [-16.611 to -7.551] | <0.001* |
| Pre-procedure furosemide injection | 16.303 [12.172 to 20.434] | <0.001* |  | 17.715 [12.991 to 22.440] | <0.001* |  | 17.596 [12.857 to 22.335] | <0.001* |  | 22.477 [16.736 to 28.217] | <0.001* |
| Pre-procedure dopamine use | 5.056 [1.729 to 8.383] | 0.003* |  | 6.702 [2.815 to 10.589] | 0.001* |  | 6.677 [2.784 to 10.570] | 0.001* |  | 4.404 [-0.087 to 8.895] | 0.055 |

The NRS-2002 scores already took age into account, and thus age was not adjusted in the multivariable model. Abbreviations refer to Table 1.

*P <0.05

**Table S4.** Multivariable logistic regression models on CI-AKI

|  | NRS-2002 |  |  | CONUT |  |  | PNI |  |  | GNRI |  |
| --- | --- | --- | --- | --- | --- | --- | --- | --- | --- | --- | --- |
|  | OR [95% CI] | *P* value |  | OR [95% CI] | *P* value |  | OR [95% CI] | *P* value |  | OR [95% CI] | *P* value |
| Nutritional score |  |  |  |  |  |  |  |  |  |  |  |
| Quintile 1 | 1 (reference) |  |  | 1 (reference) |  |  | 2.798 [1.841 to 4.253] | <0.001 |  | 2.122 [1.447 to 3.112] | <0.001 |
| Quintile 2 | 1.330 [1.045 to 1.694] | 0.021* |  | 1.043 [0.796 to 1.367] | 0.762 |  | 1.192 [0.820 to 1.733] | 0.356 |  | 1.097 [0.758 to 1.588] | 0.622 |
| Quintile 3 | 1.811 [1.341 to 2.447] | <0.001* |  | 1.401 [1.039 to 1.890] | 0.027* |  | 1.002 [0.700 to 1.434] | 0.991 |  | 1.081 [0.764 to 1.529] | 0.660 |
| Quintile 4 | 3.915 [2.678 to 5.723] | <0.001* |  | 2.473 [1.771 to 3.451] | <0.001* |  | 1 (reference) |  |  | 1 (reference) |  |
| Age, per 10 years | - | - |  | 1.074 [0.970 to 1.189] | 0.170 |  | 1.081 [0.976 to 1.198] | 0.134 |  | 1.049 [0.932 to 1.181] | 0.426 |
| Male | 0.792 [0.643 to 0.975] | 0.028* |  | 0.754 [0.609 to 0.933] | 0.009* |  | 0.764 [0.618 to 0.945] | 0.013* |  | 0.747 [0.585 to 0.954] | 0.019* |
| Diabetes | 1.155 [0.923 to 1.444] | 0.208 |  | 1.192 [0.950 to 1.496] | 0.130 |  | 1.210 [0.964 to 1.520] | 0.101 |  | 1.240 [0.954 to 1.611] | 0.108 |
| Average SBP | 0.781 [0.667 to 0.915] | 0.002* |  | 0.775 [0.660 to 0.909] | 0.002* |  | 0.767 [0.654 to 0.900] | 0.001* |  | 0.810 [0.674 to 0.974] | 0.025* |
| eGFR | 0.893 [0.786 to 1.014] | 0.080 |  | 1.045 [0.913 to 1.195] | 0.524 |  | 1.039 [0.908 to 1.189] | 0.578 |  | 0.994 [0.853 to 1.159] | 0.941 |
| Ejection fraction | 0.803 [0.702 to 0.917] | 0.001* |  | 0.787 [0.687 to 0.901] | 0.001* |  | 0.789 [0.689 to 0.905] | 0.001* |  | 0.762 [0.651 to 0.891] | 0.001* |
| Hemoglobin | 0.686 [0.582 to 0.809] | <0.001* |  | 0.694 [0.583 to 0.826] | <0.001* |  | 0.698 [0.585 to 0.832] | <0.001* |  | 0.610 [0.499 to 0.745] | <0.001* |
| C-reactive protein | 1.368 [1.223 to 1.530] | <0.001* |  | 1.211 [1.081 to 1.357] | 0.001* |  | 1.183 [1.054 to 1.328] | 0.004* |  | 1.131 [0.986 to 1.297] | 0.079 |
| Volume of contrast agent | 0.927 [0.819 to 1.049] | 0.227 |  | 0.932 [0.821 to 1.057] | 0.272 |  | 0.935 [0.824 to 1.062] | 0.303 |  | 0.822 [0.709 to 0.953] | 0.009* |
| Isotonic agent (vs. hypotonic) | 0.848 [0.683 to 1.053] | 0.136 |  | 0.924 [0.735 to 1.162] | 0.501 |  | 0.917 [0.728 to 1.154] | 0.460 |  | 0.995 [0.761 to 1.299] | 0.968 |

Multivariable logistic regression adjusted for age (per 10 years), gender (male or female), diabetes (yes or no), average SBP (<90, 90-114, 115-139, ≥140 mmHg), eGFR (<30, 30-59, 60-89, ≥90 ml/min/1.73m^2^), ejection fraction (<50, 50-64, ≥65 %), hemoglobin (<110, 110-139, ≥140 g/L), C-reactive protein (<5, 5-10, ≥10 mg/L), volume of contrast agent consumption (<60, 60-119, ≥120 mg), and type of contrast agent (isotonic or hypotonic). The NRS-2002 scores already took age into account, and thus age was not adjusted in the multivariable model. Abbreviations refer to Table 1. *P <0.05

**Table S5.** Multivariable logistic regression models on CI-AKI (with additional adjustment for medications)

|  | NRS-2002 |  |  | CONUT |  |  | PNI |  |  | GNRI |  |
| --- | --- | --- | --- | --- | --- | --- | --- | --- | --- | --- | --- |
|  | OR [95% CI] | *P* value |  | OR [95% CI] | *P* value |  | OR [95% CI] | *P* value |  | OR [95% CI] | *P* value |
| Nutritional score |  |  |  |  |  |  |  |  |  |  |  |
| Quintile 1 | 1 (reference) |  |  | 1 (reference) |  |  | 2.349 [1.529 to 3.610] | <0.001 |  | 1.822 [1.229 to 2.702] | 0.003 |
| Quintile 2 | 1.364 [1.067 to 1.744] | 0.013* |  | 1.059 [0.806 to 1.392] | 0.682 |  | 1.068 [0.730 to 1.561] | 0.735 |  | 1.009 [0.693 to 1.469] | 0.961 |
| Quintile 3 | 1.790 [1.319 to 2.430] | <0.001* |  | 1.308 [0.964 to 1.773] | 0.084 |  | 0.960 [0.668 to 1.379] | 0.825 |  | 1.035 [0.728 to 1.469] | 0.849 |
| Quintile 4 | 4.026 [2.732 to 5.932] | <0.001* |  | 2.230 [1.586 to 3.136] | <0.001* |  | 1 (reference) |  |  | 1 (reference) |  |
| Age, per 10 years | - | - |  | 1.081 [0.976 to 1.198] | 0.137 |  | 1.092 [0.985 to 1.210] | 0.096 |  | 1.064 [0.944 to 1.199] | 0.308 |
| Male | 0.808 [0.654 to 0.997] | 0.047* |  | 0.782 [0.630 to 0.971] | 0.026* |  | 0.792 [0.639 to 0.982] | 0.034* |  | 0.788 [0.614 to 1.011] | 0.061 |
| Diabetes | 1.148 [0.913 to 1.443] | 0.237 |  | 1.205 [0.956 to 1.520] | 0.115 |  | 1.217 [0.964 to 1.536] | 0.098 |  | 1.279 [0.978 to 1.671] | 0.072 |
| Average SBP | 0.852 [0.724 to 1.002] | 0.053 |  | 0.861 [0.730 to 1.015] | 0.075 |  | 0.851 [0.722 to 1.003] | 0.055 |  | 0.920 [0.760 to 1.113] | 0.393 |
| eGFR | 0.921 [0.809 to 1.049] | 0.214 |  | 1.078 [0.940 to 1.237] | 0.282 |  | 1.073 [0.935 to 1.232] | 0.316 |  | 1.021 [0.873 to 1.192] | 0.798 |
| Ejection fraction | 0.949 [0.821 to 1.097] | 0.478 |  | 0.938 [0.810 to 1.087] | 0.397 |  | 0.938 [0.809 to 1.087] | 0.396 |  | 0.886 [0.749 to 1.047] | 0.156 |
| Hemoglobin | 0.701 [0.594 to 0.828] | <0.001* |  | 0.704 [0.590 to 0.840] | <0.001* |  | 0.702 [0.587 to 0.838] | <0.001* |  | 0.621 [0.507 to 0.761] | <0.001* |
| C-reactive protein | 1.288 [1.149 to 1.444] | <0.001* |  | 1.141 [1.015 to 1.283] | 0.028* |  | 1.118 [0.992 to 1.259] | 0.067 |  | 1.071 [0.930 to 1.233] | 0.340 |
| Volume of contrast agent | 0.962 [0.838 to 1.104] | 0.581 |  | 0.925 [0.804 to 1.064] | 0.276 |  | 0.921 [0.801 to 1.061] | 0.255 |  | 0.823 [0.699 to 0.969] | 0.019* |
| Isotonic agent (vs. hypotonic) | 0.837 [0.671 to 1.043] | 0.114 |  | 0.891 [0.705 to 1.126] | 0.334 |  | 0.883 [0.698 to 1.117] | 0.299 |  | 0.965 [0.734 to 1.267] | 0.796 |
| Pre-procedure statin | 0.557 [0.433 to 0.717] | <0.001* |  | 0.650 [0.507 to 0.833] | 0.001* |  | 0.672 [0.523 to 0.863] | 0.002* |  | 0.612 [0.464 to 0.809] | 0.001* |
| Pre-procedure furosemide | 2.234 [1.727 to 2.890] | <0.001* |  | 2.336 [1.793 to 3.043] | <0.001* |  | 2.304 [1.767 to 3.004] | <0.001* |  | 2.415 [1.751 to 3.330] | <0.001* |
| Pre-procedure dopamine | 1.200 [0.949 to 1.518] | 0.128 |  | 1.374 [1.077 to 1.753] | 0.011* |  | 1.400 [1.096 to 1.788] | 0.007* |  | 1.407 [1.052 to 1.881] | 0.021* |

Multivariable logistic regression adjusted for age (per 10 years), gender (male or female), diabetes (yes or no), average SBP (<90, 90-114, 115-139, ≥140mmHg), eGFR (<30, 30-59, 60-89, ≥90ml/min/1.73m^2^), ejection fraction (<50, 50-64, ≥65%), Hemoglobin (<110, 110-139, ≥140g/L), C-reactive protein (<5, 5-10, ≥10mg/L), volume of contrast agent consumption (<60, 60-119, ≥120mg), and type of contrast agent (isotonic or hypotonic), pre-procedure statin use (yes or no), pre-procedure furosemide injection (yes or no), and pre-procedure dopamine use (yes or no). The NRS-2002 scores already took age into account, and thus age was not adjusted in the multivariable model. Abbreviations refer to Table 1. *P <0.05
